# Supplementary material for: Formation of a single quasicrystal upon collision of multiple grains
Source: Nat Commun. 2021 Oct 11;12:5790. doi: 10.1038/s41467-021-26070-9 (PMC8505427; doi:10.1038/s41467-021-26070-9)
Supplement: Supplementary file 1 — Supplementary Information [file 41467_2021_26070_MOESM1_ESM.pdf]

Supplementary information

Formation of a single quasicrystal upon collision of multiple grains

I. Han et al.

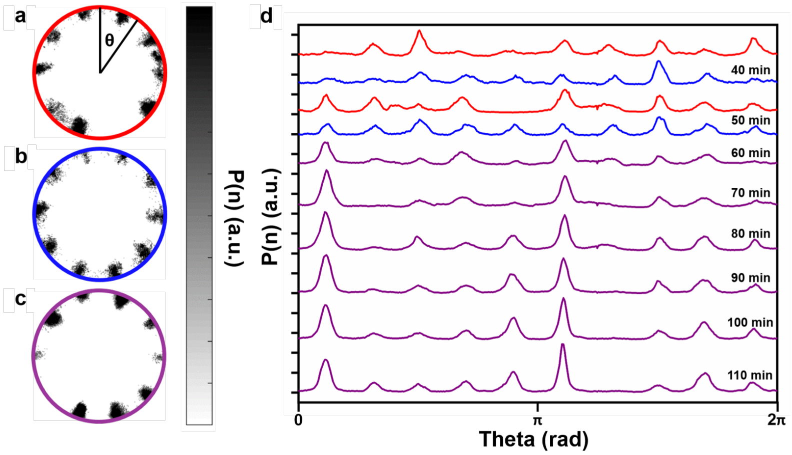

Supplementary Fig. 1: **Distribution of facet orientations.** Detailed view of stereographic projections of interface (facet) orientations of d-QC seeds on the (a) left-hand-side, (b) right-hand-side in Fig. 1(b) after 50 min of cooling and (c) the coalesced d-QC after 110 min of cooling. Zone axis of projections is the specimen $\hat{z}$ direction, which corresponds to <00001> in all cases. It follows that the QCs in (a,b) possess parallel long axes and small (<1°) misorientation in the aperiodic plane. $\boldsymbol{P(n)}$ represents the probability (weighted by area fraction) of finding an interfacial normal, $\boldsymbol{n}$, along a particular direction. Peaks in the distribution indicate a highly anisotropic or faceted structure. In principle, a facet should have a single (discrete) orientation. Yet the peaks have finite width, likely a result of mesh smoothing. (d) Radial distribution of facet orientations obtained from 40 min to 110 min. The red, blue, and purple colors represent the d-QC seeds on left- and right-hand-side (before impingement) and the coalesced d-QC, respectively. Angular measurements start at the 12 o'clock position of the stereographic projection and increase clockwise. Two facets (peaks) are separated by an angle of nearly 36°, which is consistent with a decaprismatic morphology of the d-QC phase.

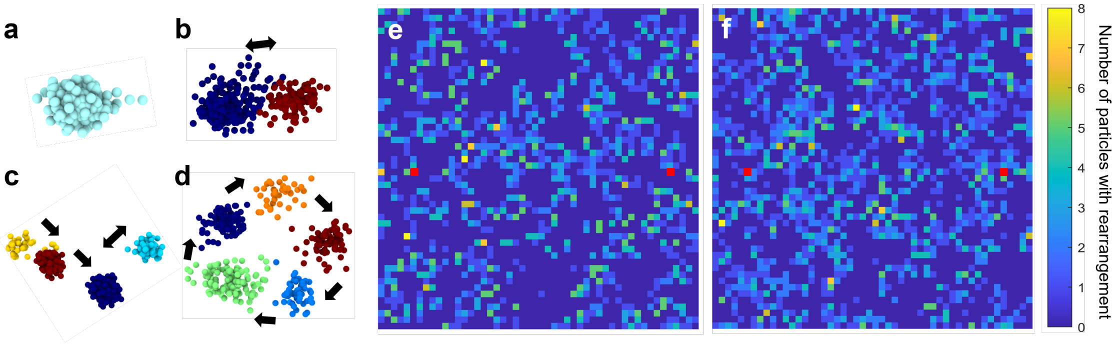

Supplementary Fig. 2: **Particle trajectory analysis.** Trajectories of a single particle in a solid, decagonal QC. The viewpoint is parallel to the periodic ⟨00001⟩ direction. Gaussian mixture model [1] and clustering algorithm [2] are used to classify the types of particle motion in 2D, e.g., (a) vibration, (b) oscillating phason flip, (c) multiple particle flips, and (d) cyclical motion on the pentagon vertices. Individual particle flips and coordinated particle flips [3] are involved in (b-d), i.e., they may be identified if more than one cluster is detected (note each cluster is depicted in a single, unique color). The number of particles that re-order by particle flips is counted in each pixellated region of the simulations with (e) fixed seeds and (f) non-fixed seeds, in order to map the number density of particles showing phason flips. The red pixels in (e,f) represent the two QC seeds (the initial misorientation between them is zero). The number density of particles that flip is relatively uniform over the simulation domain in both cases.

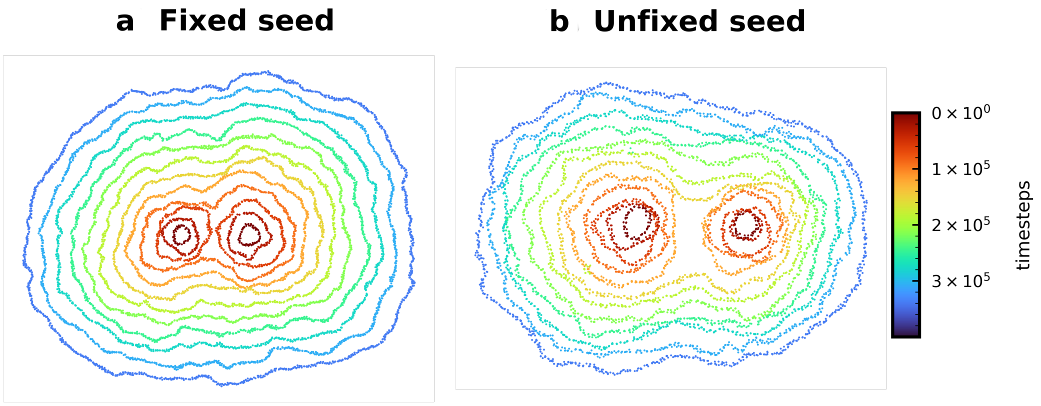

Supplementary Fig. 3: **Solid-liquid interface in simulation.** Solid-liquid interfaces over 4×10^5 simulation time steps for the case of (a) fixed seeds ($\theta = 9°$, $L = 40d$) and (b) unfixed seeds. To simulate, two seeds with $\theta = 9°$ and $L = 40d$ were placed in the fluid. For the (a) fixed seed case, seeds positions were fixed for the entire simulation. Particles in the (b) unfixed seed simulation were allowed to move freely following nucleation. Interfaces are colored according to time step. Differences in collision time between the two systems is likely stochastic.

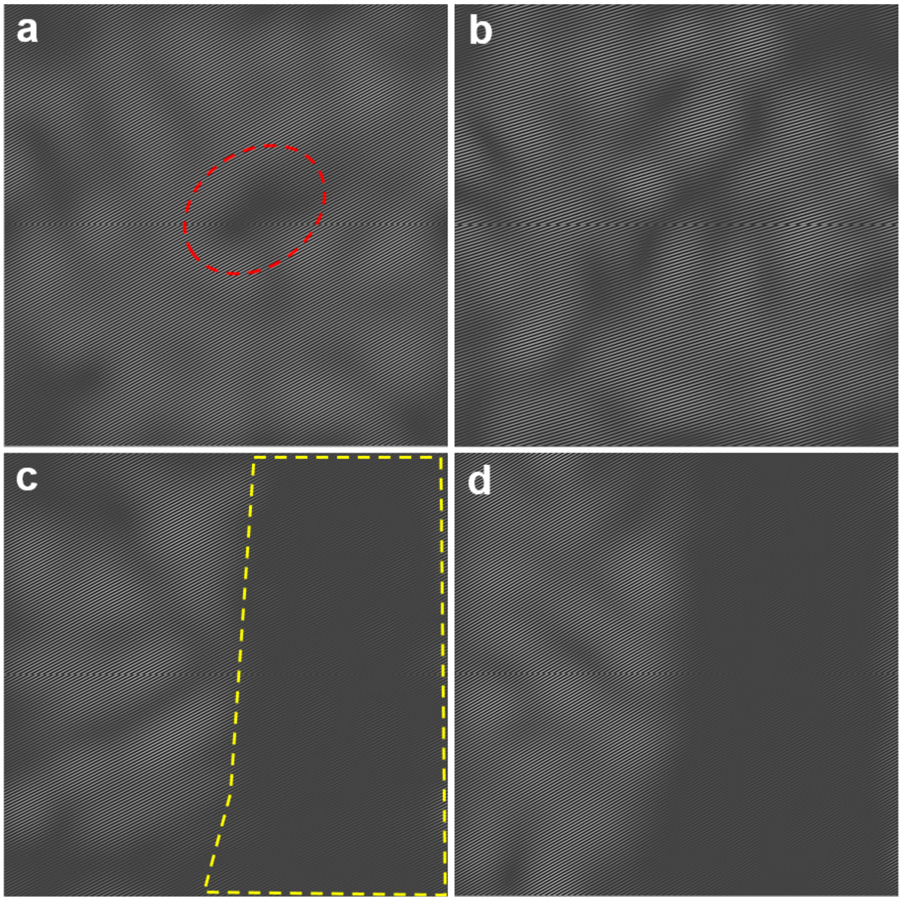

Supplementary Fig. 4: **Density modes from simulation.** Density modes associated with one pair of Bragg peaks (one basis vector and its negative) for (a) 3°, (a) 9°, (c) 10°, and (d) 15° initial misorientations. (a-d) corresponds to the diffraction patterns in Fig. 3(a-d). The presence of partially different contrast in (a-d) indicates regions with local phonon strain as highlighted in red in (a). On the other hand, the distinctive contrast in (c,d) is associated with different grain orientations, as highlighted in yellow in (c).

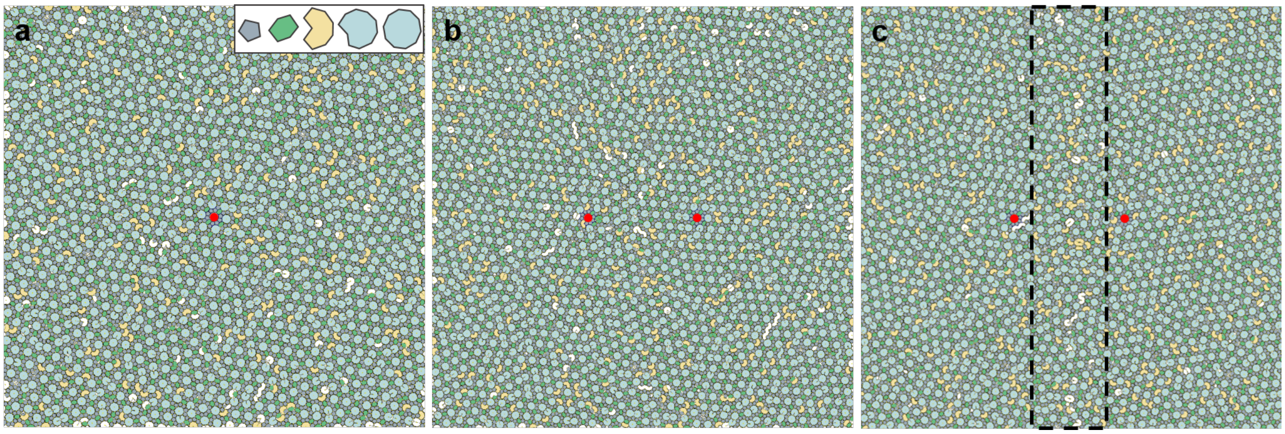

Supplementary Fig. 5: **Decagonal tilings.** Tilings calculated at ~ 2.5×10^7 simulation timesteps, of (a) single QC growth, and growth from two QCs with (b) 9° and (c) 18° misorientations. We classified five tiles [4] into four classes (see inset in (a)). From largest to smallest, the four classes are colored in light blue, yellow, green, and grey. The red tiles indicate the initial QC seed positions. Images are cropped from the full volume for better visualization. (a) and (b) demonstrate few, dispersed tiling violations (white regions that are not assigned to tiles), whereas tiling violations in (c) are concentrated along the grain boundary region (boxed in black). These findings support the formation of a single QC and a grain boundary, respectively.

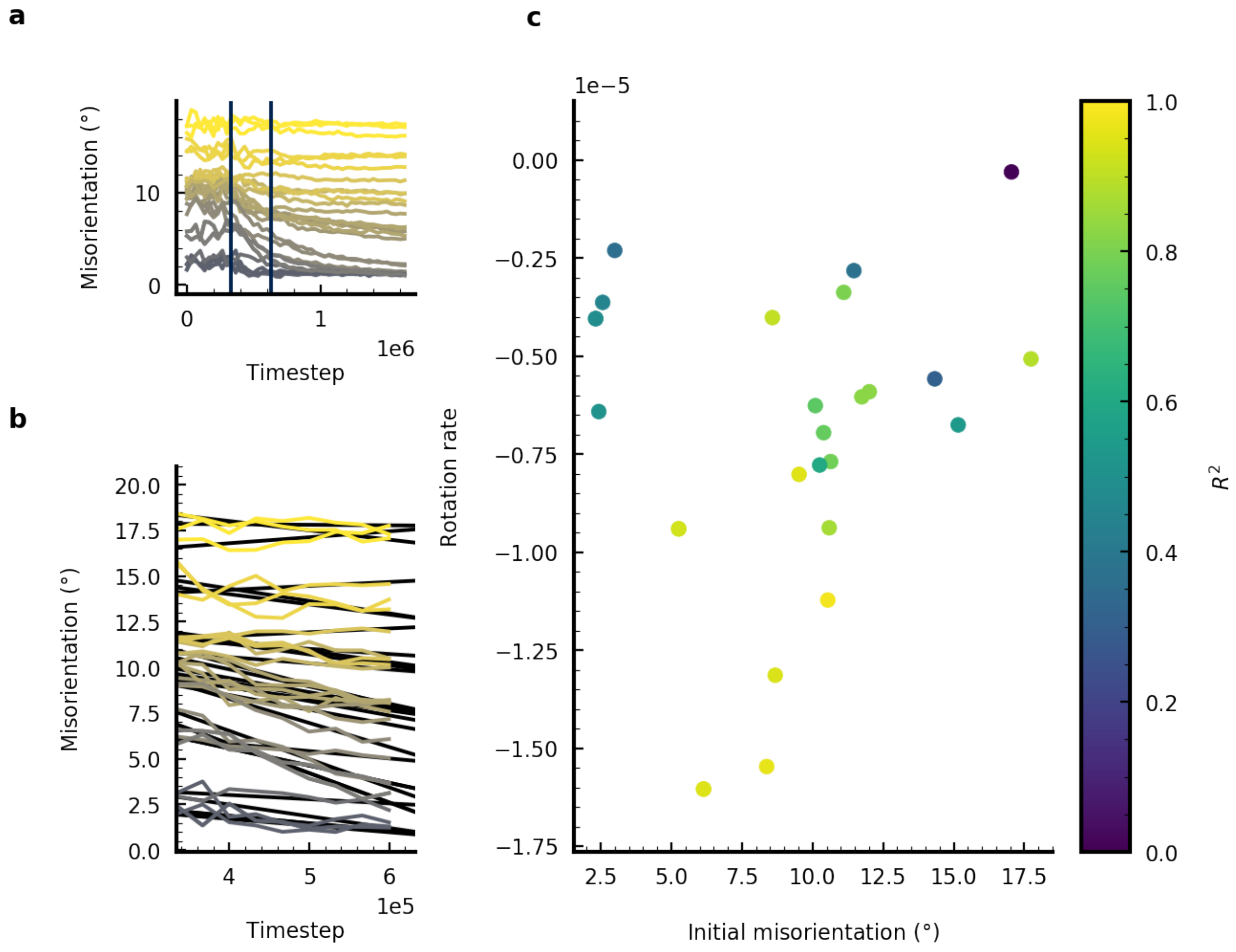

Supplementary Fig. 6: **Rotation rates of decagonal quasicrystals.** Misorientations between d-QC grains during growth in simulation. Here, (a) shows the change in misorientation between grains over 1 million timesteps, where timesteps are expressed as dimensionless time, τ, and $t = 0$ indicates the start of the simulation. We may estimate each timestep, τ, to be in the order of magnitude of 10^{-13} to 10^{-12} seconds. Lines are colored according to seed misorientation, where yellow (light grey) indicates large misorientation and grey indicates small misorientation. Grain rotation begins when grain collision occurs around $\sim 3 \times 10^5$ timesteps, then continues with a linear trend until $\sim 6 \times 10^5$ timesteps. This region of linear grain rotation is indicated between dark blue lines on (a) and expanded in (b). In (b), yellow and grey lines correspond to the same seed misorientations as in (a). We use linear regression to model rotation in each simulation (black lines), and determine rotation rates from the slope of each line. In (c), we plot rotation rate as a function of average grain misorientaion before collision. We use average grain misorientation before collision, rather than seed misorientation because there are small fluctuations in grain orientation during growth. These fluctuations are observed even in growth of single grains and can drastically affect the rotation rates when misorientation is near $\Delta\theta_{crit}$. Rotation rates are colored according to the linear correlation coefficient, $\boldsymbol{R}^2$, where $\boldsymbol{R}^2 = 1$ is yellow (light grey) and $\boldsymbol{R}^2 = 0$ is purple (dark grey). Here, we observe faster rotation as initial misorientation increases from $\sim 2.5°$ to $\sim 6°$. Then, as misorientation approaches $\Delta\theta_{crit} \approx 9°$, we observe a rapid decrease in the magnitude of the rotation rate. These results are consistent with a driving force of $\gamma'_{gb} = \frac{d\gamma_{gb}}{d\Delta\theta}$, where γ_{gb} is grain boundary energy and $\Delta\theta$ is grain misorientation. Typically, γ_{gb} increases with $\Delta\theta$ until it reaches some critical point ($\Delta\theta_{crit}$), above which λ is a much weaker function of $\Delta\theta$. This means we should expect the driving force, γ'_{gb} to show a decline around $\Delta\theta_{crit}$, which is reflected in the drastic decrease in rotation rates around $\Delta\theta \approx 9°$.

Supplementary References

[1] Pedregosa, F. *et al.* Scikit-learn: Machine learning in python. *J. Mach. Learn. Res.* **12**, 2825–2830 (2011).

[2] Ramasubramani, V. *et al.* freud: A software suite for high throughput analysis of particle simulation data. *Comput. Phys. Commun.* **254**, 107275 (2020).

[3] Engel, M., Umezaki, M., Trebin, H.-R. & Odagaki, T. Dynamics of particle flips in two-dimensional quasicrystals. *Phys. Rev. B* **82**, 134206 (2010).

[4] Engel, M. & Trebin, H.-R. Stability of the decagonal quasicrystal in the lennard–jones–gauss system. *Phil. Mag.* **88**, 1959–1965 (2008).
